# Relative Contributions of the Speed Characteristic and Other Possible Ecological Factors in Synchronization to a Visual Beat Consisting of Periodically Moving Stimuli

**DOI:** 10.3389/fpsyg.2018.01226

**Published:** 2018-07-18

**Authors:** Yingyu Huang, Li Gu, Junkai Yang, Shengqi Zhong, Xiang Wu

**Affiliations:** Department of Psychology, Sun Yat-Sen University, Guangzhou, China

**Keywords:** ecology, music, sensorimotor, timing, visual

## Abstract

Daily music experience involves synchronizing movements in time with a perceived periodic beat. Contrary to the auditory-specific view of beat synchronization, synchronization to a visual beat composed of a periodically bouncing ball has been shown to be not less stable than synchronization to auditory beats. The ecological relevance of periodically moving visual stimuli is considered to be essential for such synchronization improvement. However, multiple factors could be associated with the ecological relevance and the relative contributions of the ecological factors to the synchronization improvement remain unclear. The present study investigated whether ecological factors other than a proposed critical factor, i.e., the speed characteristic, are required to account for the synchronization improvement of the bouncing ball. A periodically contracting ring that had the same speed characteristic as the periodically bouncing ball but lacked other possible ecological factors of the ball was designed. The results showed that synchronization was more stable for the bouncing ball than for the contracting ring, and this stability difference was larger in the difficult 300-ms than in the comfortable 600-ms inter-beat interval tapping condition. The finding suggests that ecological factors other than the speed characteristic are required to explain the synchronization improvement of periodically moving visual stimuli, particularly in difficult tapping conditions.

## Introduction

People often move (e.g., tap a finger or foot) in synchrony with a perceived periodic beat (or pulse) in most forms of music (Lerdahl and Jackendoff, 1983). The capacity to entrain motor behaviors to a beat is predictive (i.e., on average, taps slightly precede event onsets) and flexible (i.e., synchronization to an auditory beat is accurate for inter-onset intervals (IOI. Or inter-beat interval) ranging from 300 to 900 ms, with the most preferred IOIs being ~600 ms) (Repp, 2006). One key feature of beat synchronization is the auditory advantage; synchronization is less stable to a visual (e.g., flashes of a light) than to an auditory beat (e.g., an auditory metronome) (Repp, 2006). According to this modality bias in beat synchronization performance, the auditory-specific view of beat synchronization suggests tighter connections between the auditory and motor cortices than between the visual and motor cortices for beat synchronization (Thaut et al., 1999; Zatorre et al., 2007; Patel, 2014). However, the auditory specificity of beat synchronization has recently been challenged by studies employing periodically moving visual stimuli. Synchronization to a visual beat can be improved using periodically moving stimuli instead of a conventional flashing light (Hove et al., 2010, 2013a; Iversen et al., 2015). In particular, synchronization to a periodically bouncing ball with a uniformly varying speed was found to be not less stable than that to an auditory beat (Gan et al., 2015).

These advances employing periodically moving visual stimuli suggest that beat synchronization could be non-specific to the auditory modality, and an essential question to be addressed is by which mechanisms moving visual stimuli improve beat synchronization. The employment of periodically moving visual stimuli in beat synchronization may be initially proposed by Repp and Penel, who suggested that moving visual stimuli are more often experienced in our environment: “A flashing light is not a common visual experience, whereas moving objects and organisms are ubiquitous” (page 268) (Repp and Penel, 2004). Hove et al. (2013a) also stated that compared with moving visual stimuli, visual flashes “lack ecological validity” (page 314). Studies used an up-down bar periodically moving with a constant speed found that synchronization to the bar was more stable than synchronization to visual flashes, but was still less stable than synchronization to auditory tones (Hove et al., 2010, 2013a). Iversen et al. (2015) employed a periodically bouncing ball that had a speed varied according to a rectified sinusoid, and showed that synchronization with the bouncing ball was close to synchronization with auditory tones. More recently, Gan et al. (2015) used a periodically bouncing ball that had a uniformly varying speed (i.e., simulating the effect of gravity) and found that synchronization to the bouncing ball was not less stable than synchronization to auditory tones. These results therefore support a role of ecological relevance in synchronization to a visual beat (Repp and Penel, 2004; Hove et al., 2013a) and suggest that the speed characteristic could be a critical factor for the synchronization improvement by periodically moving visual stimuli as compared to visual flashes (Hove et al., 2013a; Gan et al., 2015; Iversen et al., 2015).

Despite of these findings, the ecological relevance in synchronization to a visual beat requires further investigation. Take the periodically bouncing ball with a uniformly varying speed as an example, which is so far the most effective stimulus type in improving synchronization to a visual beat (Gan et al., 2015). Compared with static flashes, the bouncing ball has spatiotemporal changes which lead to a collision point, and would be easier for people to detect and move with (Hove et al., 2010, 2013a); compared with periodically moving visual stimuli with a constant speed, the bouncing ball has a varying speed which results in a peak speed at the collision point, and would yield more realistic movement (Hove et al., 2010; Su, 2014; Gan et al., 2015; Iversen et al., 2015); and compared with the periodically bouncing ball that has a speed varied according to a rectified sinusoid, the bouncing ball with a uniformly varying speed simulates the effect of gravity and thus may be more natural to the subjects (Gan et al., 2015). As introduced above, the superiority of the bouncing ball with a uniformly varying speed over flashes and other types of periodically moving visual stimuli suggests the importance of the speed characteristic in synchronization to a visual beat. However, besides the speed characteristic, other possible factors could also be associated with the ecological relevance of the bouncing ball and contribute to the effect of the bouncing ball (i.e., the substantial improvement of synchronization to a visual beat by the bouncing ball as compared with static flashes). Whereas the speed characteristic is considered to be critical, it remains to be clarified how critical the speed characteristic is. In other words, to what extent the speed characteristic could account for the overall effect of the bouncing ball? Are other possible ecological factors required? To answer this question, one way could be to identify other possible ecological factors and to examine individual contributions of these factors to the bouncing ball effect as compared with the contribution of the speed characteristic. Such efforts have been made, e.g., in the study of Gan et al. (2015) in which movement smoothness was examined and showed weak influence on synchronization performance. For this investigation manner, the difficulty exists due to the factor that many factors other than the speed characteristic could be associated with the ecological relevance of the bouncing ball. For instance, a ball may be a more naturally bouncing object compared with a bar, and a basketball would be more realistic than a uniform color ball. The collision of a ball on a surface (e.g., the floor) may imply a sense of the sound of collision which would potentially facilitate synchronization, and the collision sound might also be related to the type and speed of the bouncing object (i.e., the collision sounds may be different between a ball and a bar, and between objects with or without a peak speed). It would be hard (perhaps impossible) to list all possible factors that are related to why the bouncing ball is such an ecological stimulus type, which makes it difficult to further understand the relative contributions of the speed characteristic and other possible factors, particularly in terms of the overall effect of the bouncing ball.

**Figure 1:**
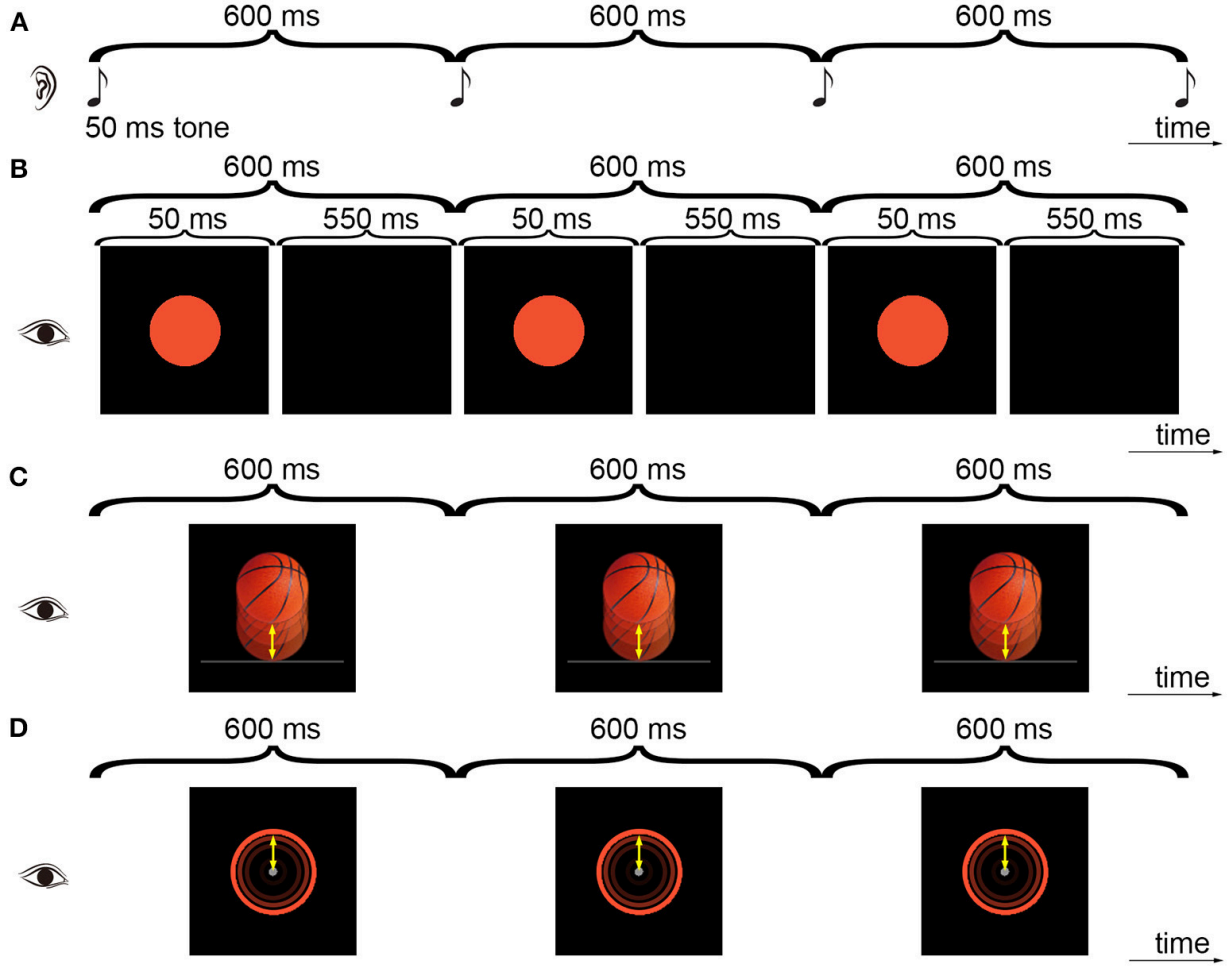
Illustration of the experimental stimuli. The subjects tapped along with an auditory tone sequence **(A)**, a visual flashing ball sequence **(B)**, a visual bouncing ball sequence **(C)**, or a visual contracting ring sequence **(D)**. Three cycles of the 600-ms IOI sequences from Experiment 1 are shown. All the points on the ring had a varying speed as the bouncing ball and the movement was indicated by the arrows (In **D**, the movement of the point on the top of the ring is indicated).

Given the above introduced difficulty in investigating the relative contributions of the speed characteristic and other possible ecological factors to the effect of ecological relevance in improving synchronization to a visual beat, the present study tried another investigation manner. For an ecological stimulus type such as the periodically bouncing ball with a uniformly varying speed, we asked whether it is possible to construct a periodically moving visual stimulus that is less ecologically relevant than the bouncing ball but has the same speed characteristic of the bouncing ball. Such a design will not resolve the issue of identifying possible ecological factors other than the speed characteristic, but would be helpful in investigation of the relative contributions of the speed characteristic and other possible factors. Specifically, if synchronization to such a stimulus was as stable as synchronization to the bouncing ball, the speed characteristic would be sufficient to explain the effect of ecological relevance. If synchronization was more stable for the bouncing ball than for such a stimulus, other possible factors would be required to account for the effect of ecological relevance. To this end, in Experiment 1 we devised a 600-ms IOI visual beat that was composed of a ring periodically contracting to a central collision point (Figure 1D). All the points on the contracting ring had a varying speed with the peak speed at the collision point, which was the same as the periodically bouncing ball (Gan et al., 2015) (Figure 1C). The contracting ring had the same speed characteristic as the bouncing ball but lacked other possible factors associated with the ecological relevance of the bouncing ball. Moreover, because beat synchronization is known to be stable for auditory tones and unstable for visual flashes (Repp, 2005), as in previous bouncing ball studies (Gan et al., 2015; Iversen et al., 2015), an auditory beat composed of auditory tones (Figure 1A) and an visual beat composed of visual flashes (Figure 1B) were involved in the present study to serve as references in evaluating synchronization performances of the bouncing ball and contracting ring.

For two considerations, Experiment 2 was carried out which was the same as Experiment 1 with the exception that a short 300-ms IOI was used. First, Experiment 2 was conducted to examine a possibility that the contribution of the speed characteristic to the effect of the periodically bouncing ball, relative to other possible ecological factors, may change in different tapping conditions. The short 300-ms IOI is a difficult tapping condition (Repp, 2005), especially for tapping to a visual beat (Gan et al., 2015). In Gan et al. (2015), the subjects verbally described the 300-ms IOI bouncing ball as “unnaturally fast.” Therefore, whereas the speed characteristic is considered to be the most critical factor for longer IOIs, it may no longer be critical for the 300-ms IOI and other possible factors may make more contributions to the effect of the bouncing ball. More specifically, if synchronization was more stable for the bouncing ball than for the contracting ring in the 600-ms IOI condition (which would indicate that factors other than the speed characteristic are required to interpret the effect of the bouncing ball), a larger performance difference between the two stimulus types was supposed to be observed in the 300-ms IOI condition (because the speed characteristic would make less contribution relative to the other possible factors). Second, Experiment 2 was performed to test the effect of a confounding factor regarding the design in Experiment 1. It has been observed that synchronization performance of a periodically bouncing ball or a periodically moving bar deteriorates when the stimulus moves horizontally, suggesting that synchronization is influenced by the compatibility between the direction of motion of the moving stimulus (up-down for vertical movement and left-right for horizontal movement) and the direction of motion of the tapping finger (up-down) (Hove et al., 2010; Gan et al., 2015). The overall movement direction of the contracting ring was inward-outward, which was also inconsistent with the up-down movement of the tapping finger (though the incompatibility may not be as strong as that for the left-right stimulus movement). Accordingly, if synchronization was less stable for the contracting ring than for the bouncing ball, the effect may also be explained by the compatibility factor. However, if the performance difference between the bouncing ball and the contracting ring was larger in the 300-ms than in the 600-ms IOI condition, the result would be hard to be explained by the compatibility factor because the compatibility factor did not change in the 600- and 300-ms IOI conditions.

Moreover, two control experiments were conducted to investigate the influence of the movement smoothness of a periodically moving visual stimulus on synchronization performance. In the pre-testing of Experiment 2, one author (YJK) noticed that for the 300-ms IOI, the movement of the contracting ring was not as smooth as that of the bouncing ball, in particular around the collision point; i.e., clear movement discontinuities could be observed when the contracting ring was close to the collision point. This movement discontinuity is related to a ratio between the movement distance and the size of the periodically moving visual object (Gan et al., 2015). For the same movement distance, the ratio was small for the bouncing ball whereas was large for the thin contracting ring. This would result in large discontinuities for the contracting ring, especially in the short 300-ms IOI condition. The related concern was that the results in Experiments 1 and 2 could be explained by the difference in the movement discontinuity between the bouncing ball and contracting ring. If higher synchronization stability for the bouncing ball than for the contracting ring was observed, the result may be due to the large movement discontinuity of the contracting ring; and if greater stability difference between the two stimulus types in the 300-ms than in the 600-ms IOI condition was observed, the result may be due to the fact that the discontinuity difference between the two stimulus types was larger in the short 300-ms IOI condition. This concern was addressed in two control experiments, which manipulated the movement-distance/object-size ratio and examined whether the change of the ratio could explain the stability difference between the two stimulus types.

## Materials and methods

### Participants

Fifteen subjects (all right-handed, one male, mean age ± *SD* 21.80 ± 2.81) participated in Experiments 1 and 2, 10 subjects (all right-handed, three male, mean age ± *SD* 23.00 ± 2.06) participated in Control experiment 1, and 15 subjects (all right-handed, four male, mean age ± *SD* 23.00 ± 2.06) participated in Control experiment 2. Three subjects in Experiments 1 and 2 (playing instruments including Erhu, piano, or keyboard for 5–8 years) and two subjects in Control experiment 1 (playing piano for 5 or 10 years) reported musical experience. Two subjects in Experiments 1 and 2 (playing video games or basketball for 5–6 years), and one subject in Control experiment 2 (playing video games for 6 years) reported special visual experience. All subjects had normal hearing and had normal or corrected-to-normal vision. The research protocols in this study were approved by the Institutional Review Board of Psychology Department of Sun Yat-Sen University. The methods were carried out in accordance with the relevant guidelines and regulations. All subjects gave written informed consent.

### Power analysis

As introduced above, in a beat synchronization task the performance of the visual bouncing ball with a uniformly varying speed was much greater than that of visual flashes and was not poorer than that of auditory tones (Gan et al., 2015). The question asked in the present study was whether the performance of the periodically contracting ring could be comparable to that of the periodically bouncing ball. The improvement of synchronization to a visual beat by the bouncing ball is characterized by the substantial improvement of performance by the visual bouncing ball as compared with visual flashes, which was thus referred to as the crucial effect tested in the current power analysis. A priori power analysis was performed using G^*^Power 3 (Faul et al., 2007) to examine the required sample size to detect the crucial effect in the present study. The size of the crucial effect was in accordance to the effect observed in Gan et al. (2015) (stability difference between the VB and VF sequences: mean difference = 0.25; Cohen's *d*_*z*_ = 1.02). Given this effect size, the alpha level of *p* < 0.05 (two tailed), and the power of 0.8, 10 subjects were required to detect the crucial effect; which suggested that the current sample sizes would have sufficient power.

### Stimuli and procedure

The subjects sat in front of a Gimit i5 4590/GTX750Ti desktop computer with an AOC G2460PQU/BR LCD computer monitor (120-Hz refresh rate, 1920 × 1080 resolution, and 53.1 × 29.8 cm) and a HP SK-2015 or Dell KB113t computer keyboard (which introduced a systematic latency of about 10 ms in checking the tapping), and wore a PHILIPS SHM6500 headphone. The viewing distance was 50 cm. In all experiments, the subjects were asked to tap in synchrony with isochronous sequences using the index finger of their preferred hand on a key of the keyboard.

In Experiment 1, four types of isochronous sequences with a 600-ms inter-onset interval (IOI) were presented: the auditory tone sequence (AT), the visual flashing ball sequence (VF), the visual bouncing ball sequence (VB), and the visual contracting ring sequence (VR) (Figure 1). For the AT sequence, a pure tone (600 Hz, 50-ms duration) was presented every 600 ms. The tone was delivered at 65 dB SPL, which was the same for all subjects. An orange ball with 1.74 cm (65 screen pixels) in diameter was displayed at the center of the computer screen with a black background. The subjects were required to fixate on the ball and to maintain attention on the auditory task. For the VF sequence, the ball flashed every 600 ms (the ball lasted for 50 ms and disappeared for the remaining IOI time). For the VB sequence, the ball was replaced with a realistic basketball, which continually moved 0.92 cm (movement distance) down to touch a bar (3.54 × 0.06 cm. The height corresponded to 2 pixels: pixels 573 and 574 in y-axis of the screen. There was 1 pixel overlap of the ball and the bar upon impact, with the bar presented in the front) and then moved up to the initial position. The periodically bouncing ball had a uniformly varying speed with the acceleration of 0.20 m/s^2^. Each movement step lasted for a frame (Table 1). The constructions of the AT, VF, and VB sequences have been described in detail in our previous work (Gan et al., 2015; Huang et al., 2017; Mu et al., 2017). For the VR sequence, an orange ring (0.1 cm thickness; corresponding to 4 pixels) continually contracted 0.92 cm to the center (as a disk with a 0.2 cm diameter at the center) and then expanded back to the initial position. Same as the bouncing ball, the periodically contracting ring had a uniformly varying speed with the acceleration of 0.20 m/s^2^. Thus, all the points on the ring had the same speed as the bouncing ball. A gray central fixation point with a 0.2 cm diameter was continually displayed during the VR sequence. The stimuli were presented using Psychtoolbox 3.0.12 for Matlab (http://psychtoolbox.org) running on Windows 7 (http://www.microsoft.com). The setup and programming of Psychtoolbox in the present study followed the procedures as recommended on the Psychtoolbox website to archive precise timing of stimulus presentation [e.g., double buffer for visual presentation and ASIO for audio presentation. For more details, see (Kleiner et al., 2007) and http://psychtoolbox.org]. Moreover, the program was set to run under the highest priority to thereby avoid interrupts by other background processes. The durations of visual stimuli and the intervals were presented in terms of frames, i.e., as a multiple of frames. For example, the 50-ms duration of the flash was equivalent to 50/(1000/120) = 6 frames and the 600-ms interval was equivalent to 600/(1000/120) = 72 frames. The bouncing ball and contracting ring were presented frame-by-frame, i.e., each movement step lasted for a frame. MATLAB R2010b (The Mathworks, Natick, MA, USA) was used to present stimuli, collect data, and analyze data. Event onsets referred to the onsets of the auditory tone, the onsets of the visual flashes, the moment when the ball touched the bar, or the moment when the ring contracted to the center. Each sequence had 55 events (54 IOIs or circles). Each sequence type was repeated six times and was followed by a 20-s rest. Stimulus presentation was self-paced, that is, the subjects pressed the space bar to start a sequence. The order of the sequence types was counterbalanced across the subjects. The subjects were instructed to tap in synchrony with the tones in the AT sequence, the flashing balls in the VF sequence, the moments when the bouncing ball moved to the lowest position (i.e., touching the bar) in the VB sequence, and when the contracting ring contracted into a disk (i.e., touching the center point) in the VR sequence.

**Table 1:** Vertical position (in screen pixel) of the bottom edge of the bouncing ball for each movement step.

**Step**	**Position**	**Step**	**Position**	**Step**	**Position**	**Step**	**Position**
1	538	19	547	37	573	55	547
2	538	20	548	38	571	56	546
3	538	21	549	39	569	57	545
4	538	22	550	40	567	58	544
5	539	23	551	41	566	59	543
6	539	24	552	42	564	60	543
7	539	25	554	43	562	61	542
8	539	26	555	44	561	62	541
9	540	27	556	45	559	63	541
10	540	28	558	46	558	64	540
11	541	29	559	47	556	65	540
12	541	30	561	48	555	66	539
13	542	31	562	49	554	67	539
14	543	32	564	50	552	68	539
15	543	33	566	51	551	69	539
16	544	34	567	52	550	70	538
17	545	35	569	53	549	71	538
18	546	36	571	54	548	72	538

*The trajectory of the bouncing ball for the 600-ms IOI (i.e., one cycle, including a down phase and an up phase) had 72 movement steps (Step 37 represented the collision point) and each step lasted for a frame. For each step, the vertical position of the bottom edge of the ball is shown, in terms of the screen pixel. Note that while realistic motion in nature is continuous, displaying the motion on a screen is inevitably discrete, limited by the temporal and spatial resolutions of the screen. Therefore, the current bouncing ball had 72 steps (i.e., limited by the temporal resolution) and the position of each step was according to a pixel (i.e., limited by the spatial resolution). Accordingly, when the ball was supposed to move slowly, distances between adjacent steps could be shorter than the size of a pixel and thus the ball was presented on the same screen position (e.g., Steps 1–4)*.

Experiment 2 was the same as Experiment 1 except that the IOI was 300 ms. For the VB and VR sequences, to avoid movement discontinuities around the collision point for the short 300-ms IOI, the moving distance was 0.77 cm and the acceleration was 0.68 m/s^2^ (Gan et al., 2015). Because Experiment 2 was designed to further address questions that were based on the results of Experiment 1, Experiment 2 was carried out after Experiment 1. Note that the task was a simple metronome tapping task and there was a 10-min rest between Experiments 1 and 2, thus it was unlikely that the results of Experiment 2 would be largely influenced by the effect of fatigue or training.

Control experiment 1 investigated the VB sequence using the 600- and 300-ms IOIs. The settings were the same as in Experiments 1 and 2, except that two types of diameter of the ball were used: 1.74 and 0.87 cm. Control experiment 2 investigated the VR sequence using the 600 and 300-ms IOIs. The settings were the same as in Experiments 1 and 2, except that three types of thickness (i.e., width) of the line of the ring were used: 0.1, 0.87, and 1.74 cm. (Control experiment 2 was added according to the suggestion in a discussion after Control experiment 1, thus was carried out after Control experiment 1).

### Data analyses

We used circular analysis methods because they are more suitable for the variable periodic synchronization data than the standard linear analysis methods (Fisher, 1993; Patel et al., 2009; Hove et al., 2013a). Moreover, the circular analysis methods have been suggested to be suitable for poor synchronization performance (Repp and Su, 2013; Dalla Bella and Sowinski, 2015). The analyses were performed using the CircStat toolbox (Philipp, 2009) programmed with MATLAB. The difference between the time of a tap and the time of the corresponding event onset (asynchrony) was measured by the relative phase (RP) on a unit circle (–pi to pi. 0 indicated perfect alignment between taps and events; negative and positive values indicated taps preceding or following events, respectively; and ±pi indicated taps midway between events). Whether a sequence was successfully synchronized was assessed using the Rayleigh test of uniform distribution of the RPs. If the *p*-value of the Rayleigh test was <0.05, the null hypothesis of a uniform distribution was rejected and the alternative hypothesis of a non-uniform distribution was accepted (Hove et al., 2010). Synchronization stability was indexed by R, which was the length of the resultant (i.e., average of vectors) of the RPs and was calculated by abs(sum(exp(i^*^RP))/n) (n indicated the number of the RPs). R ranged from 0 (unstable tapping with uniformly distributed RPs) to 1 (perfectly stable tapping with a unimodal distribution of RPs). R equaled (1-circular variance). Correspondingly, mean asynchrony was indexed by the angle of the resultant of the RPs and was calculated by angle (sum(exp(i^*^RP))/n). In addition, the relation between adjacent inter-tap intervals (ITI) was assessed by the lag-1 autocorrelation of the ITIs (AC-1) (Hove et al., 2013a; Iversen et al., 2015). The positive AC-1 is characterized by successive short or successive long tap intervals and suggests that tap intervals drift away from the beat interval; and the negative AC-1 is characterized by alternating between short and long tap intervals and suggests error correction mechanisms preventing the drift (Vorberg and Wing, 1996; Hove and Keller, 2010; Hove et al., 2010; Iversen et al., 2015). For each sequence type of each subject, the stability, the mean asynchrony, and the AC-1 were calculated for individual trials and averaged across trials. The mean asynchrony and the AC-1 were only analyzed for successful trials as determined by the Rayleigh test. Because the stability *per se* is an indicator of the distribution of the RPs, both successful and unsuccessful trials were included in the stability analysis (Hove et al., 2013a,b).

In the analyses, the taps to the first five events in a sequence were omitted from the analyses because synchronization typically requires a few taps to stabilize. The asynchrony to an event with invalid taps including missing tap (i.e., there was no tap during the −1/2 to +1/2 IOI interval) and multiple taps (i.e., when there were more than one tap during the −1/2 to +1/2 IOI interval around an event) was excluded. On average, the asynchronies to 0.9 ± 2.1, 4.5 ± 4.8, 5.1 ± 4.6, and 4.1 ± 4.9 events were excluded in Experiment 1, Experiment 2, Control experiment 1, and Control experiment 2, respectively. During the analyses of the AC-1, because the computation of AC-1 requires successive taps, the ITIs for invalid taps were interpolated with their neighboring ITIs (i.e., the average of three preceding and three following ITIs) (Patel et al., 2005; Jacoby et al., 2015). In addition, in the 300-ms IOI condition, for some subjects the six trials of a sequence type were all unsuccessful in the Rayleigh test. For the analyses performed on only successful trials, if the 6 trials of a sequence type of a subject were all unsuccessful, the data of the subject were excluded (by the discussion with a statistician, the data of such poorly performed visual sequences are inappropriate to be treated as missing data in ANOVA analyses, because they were rejected by a criterion (Rayleigh test here), rather than truly missing data. This leaded to the exclusion of the data of 1 subject in Experiment 1, the data of 7 subjects in Experiment 2, the data of 2 subjects in Control experiment 1, and the data of 2 subjects in Control experiment 2. Note that the exclusion of subjects due to the Rayleigh test was not for the stability analysis, which was the primary measure in the present study and contained both successful and unsuccessful trials (as described above).

Greenhouse-Geisser corrections were applied to all ANOVA analyses. Bonferroni corrections were applied to *post-hoc t*-tests, and corrected *p*-values ≤ 0.05 were considered statistically significant. Effect sizes were reported using Cohen's *d*_*z*_ for within-subject comparisons and Cohen's *d* for between-subject comparisons (Lakens, 2013). All *t*-tests were two-tailed. ANOVAs and *t*-tests were performed using IBM SPSS Statistics 22.0 (SPSS Inc., Chicago, IL, USA).

The analyses did not contain exploratory analyses.

## Results

### Experiment 1

Beat synchronization was studied by having the subjects tap a finger along with a metronome (Repp and Su, 2013; Patel, 2014), which was composed of an isochronous sequence with a 600-ms IOI. There were four types of sequences (Figure 1): the auditory tone sequence (AT), the visual flashing ball sequence (VF), the visual bouncing ball sequence (VB), and the visual contracting ring sequence (VR). The periodically contracting ring had the same varying speed and thus the same speed characteristic as the periodically bouncing ball.

The data were analyzed using a circular analysis method in which the difference between the time of a tap and the time of the corresponding event onset was assessed by the relative phase (RP) on a unit circle (Fisher, 1993; Hove et al., 2013a). The present study focused on the analysis of synchronization stability because it has been suggested that the stability is more sensitive in identifying individual differences of synchronization performances as compared with the mean asynchrony (Hove et al., 2013a; Dalla Bella and Sowinski, 2015). Synchronization stability was indexed by R, which was the length of the resultant of the RPs (Hove et al., 2013a). R ranged from 0 (unstable tapping) to 1 (perfectly stable tapping). The mean asynchrony was calculated as the angle of the resultant of the RPs. In addition, the lag-1 autocorrelation of the inter-tap intervals (AC-1) (a negative AC-1 could suggest error correction) was also analyzed (Hove and Keller, 2010; Hove et al., 2010; Iversen et al., 2015). The Rayleigh test was used to assess whether a trial was successfully synchronized, and the percentages of such trials were reported (Hove et al., 2010). The mean asynchrony and the AC-1 were only analyzed for successful trials. Because the stability *per se* is an indicator of the distribution of the RPs, both successful and unsuccessful trials were included in the stability analysis (Hove et al., 2013a,b). The major concern of Experiment 1 was whether synchronization to the VR sequence would be as stable as synchronization to the VB sequence.

**Figure 2:**
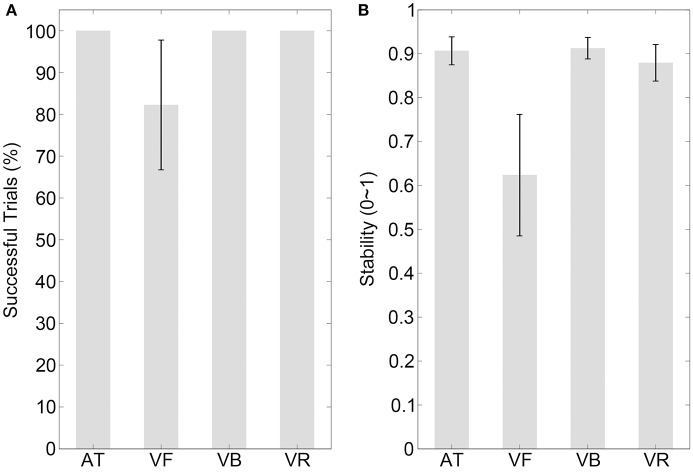
Results of experiment 1. **(A,B)** Show the percentages of successfully synchronized trials and the synchronization stabilities, respectively, for the auditory tone (AT), visual flashing ball (VF), visual bouncing ball (VB), and visual contracting ring (VR) sequences with a 600-ms IOI. Error bars indicate ±95% confidence intervals (note that the percentage of successful trials was 100% for the AT, VB, and VR sequences and the CI was not applicable).

The results of the percentage of successful trials are illustrated in Figure 2A (mean values are listed in Table 2). The percentage of successful trials was 100% for all sequence types, with the exception of the VR sequence. Comparisons between sequence types are listed in Table 3.

**Table 2:** Statistical information of synchronization measures in Experiments 1 and 2.

	**Experiment 1**				**Experiment 2**			
	**Mean**	**95% CI**	***t*-value**	***p*-value**	**Mean**	**95% CI**	***t*-value**	***p*-value**
**SUCCESSFUL TRIALS (%)**								
AT	100.00	—	—	—	93.33	[83.59, 103.08]	20.55	<0.001
VF	82.22	[66.83, 97.61]	11.46	<0.001	45.56	[21.02, 70.09]	3.98	0.001
VB	100.00	—	—	—	78.89	[61.28, 96.49]	9.61	<0.001
VR	100.00	—	—	—	53.33	[29.11, 77.55]	4.72	<0.001
**STABILITY (0**~**1)**								
AT	0.91	[0.88, 0.94]	62.21	<0.001	0.72	[0.59, 0.84]	12.34	<0.001
VF	0.62	[0.49, 0.76]	9.70	<0.001	0.27	[0.16, 0.38]	5.17	<0.001
VB	0.91	[0.89, 0.94]	82.62	<0.001	0.53	[0.39, 0.66]	8.51	<0.001
VR	0.88	[0.84, 0.92]	46.22	<0.001	0.37	[0.25, 0.50]	6.45	<0.001
**MEAN ASYNCHRONY (RADIAN)**								
AT	−0.85	[−1.14, −0.57]	−6.50	<0.001	−0.11	[−0.45, 0.23]	−0.77	>0.250
VF	−0.62	[−1.25, 0.01]	−2.13	0.053	0.03	[−0.35, 0.40]	0.17	>0.250
VB	−0.95	[−1.20, −0.69]	−8.00	<0.001	−0.01	[−0.51, 0.49]	−0.05	>0.250
VR	−0.72	[−0.92, −0.52]	−7.80	<0.001	1.03	[0.11, 1.95]	2.64	0.033
**AC-1**								
AT	−0.24	[−0.28, −0.19]	−10.71	<0.001	−0.28	[−0.51, −0.05]	−2.84	0.025
VF	−0.03	[−0.13, 0.06]	−0.75	>0.250	−0.03	[−0.27, 0.22]	−0.26	>0.250
VB	−0.31	[−0.38, −0.25]	−10.22	<0.001	−0.13	[−0.35, 0.08]	−1.49	0.181
VR	−0.25	[−0.32, −0.17]	−6.76	<0.001	0.05	[−0.11, 0.21]	0.73	>0.250

*Mean values for the percentage of successful trials, the stability, the mean asynchrony, and the lag-1 autocorrelation (AC-1) are presented. T-tests against zero were performed. (Note that when the percentage of successful trials was 100%, the CI and the t-test against zero were not applicable and are marked with “—”). Other conventions are as in Figure 2*.

**Table 3:** *Post-hoc* comparisons of synchronization measures between sequence types in Experiments 1 and 2.

	**Experiment 1**						**Experiment 2**					
	**Mean**	**95% CI**	***t*-value**	***p*-value**	**p_*corrected*_ value**	**Cohen's *dz* value**	**Mean**	**95%CI**	***t*-value**	***p*-value**	**p_*corrected*_ value**	**Cohen's *dz* value**
**SUCCESSFUL TRIALS (%)**												
AT vs. VF	17.78	[2.39, 33.17]	2.48	0.027	0.160	0.64	47.78	[18.83, 76.73]	3.54	0.003	0.020	0.91
AT vs. VB	0.00	—	—	—	—	—	14.44	[−5.25, 34.14]	1.57	0.138	>0.250	0.41
AT vs. VR	0.00	—	—	—	—	—	40.00	[17.18, 62.82]	3.76	0.002	0.013	0.97
VB vs. VF	17.78	[2.39, 33.17]	2.48	0.027	0.160	0.64	33.33	[8.18, 58.49]	2.84	0.013	0.078	0.73
VB vs. VR	0.00	—	—	—	—	—	25.56	[6.81, 44.30]	2.92	0.011	0.067	0.76
VF vs. VR	−17.78	[−33.17, −2.39]	−2.48	0.027	0.160	0.64	−7.78	[−33.85, 18.30]	−0.64	>0.250	>0.250	0.17
**STABILITY (0**~**1)**												
AT vs. VF	0.28	[0.16, 0.41]	4.85	<0.001	0.002	1.25	0.45	[0.28, 0.61]	5.81	<0.001	<0.001	1.50
AT vs. VB	−0.01	[−0.03, 0.02]	−0.45	>0.250	>0.250	0.12	0.19	[0.04, 0.33]	2.84	0.013	0.079	0.73
AT vs. VR	0.03	[−0.00, 0.06]	1.85	0.086	>0.250	0.48	0.34	[0.22, 0.47]	5.99	<0.001	<0.001	1.55
VB vs. VF	0.29	[0.16, 0.42]	4.84	<0.001	0.002	1.25	0.26	[0.12, 0.39]	4.15	0.001	0.006	1.07
VB vs. VR	0.03	[0.01, 0.06]	3.22	0.006	0.037	1.14	0.15	[0.08, 0.23]	4.39	0.001	0.004	0.53
VF vs. VR	−0.26	[−0.38, −0.13]	−4.41	0.001	0.004	0.83	−0.11	[−0.21, 0.00]	−2.06	0.059	>0.250	1.13
**MEAN ASYNCHRONY (RADIAN)**												
AT vs. VF							−0.14	[−0.57, 0.30]	−0.75	>0.250	>0.250	0.27
AT vs. VB							−0.10	[−0.81, 0.61]	−0.34	>0.250	>0.250	0.12
AT vs. VR							−1.14	[−2.16,−0.12]	−2.64	0.033	0.200	0.93
VB vs. VF							0.04	[−0.82, 0.74]	−0.11	>0.250	>0.250	0.04
VB vs. VR							−1.04	[−1.86, −0.22]	−3.00	0.020	0.120	1.06
VF vs. VR							−1.00	[−2.06,.05]	−2.25	0.059	>0.250	0.80
**AC-1**												
AT vs. VF	−0.20	[−0.29, −0.11]	−4.71	<0.001	0.002	1.26	−0.25	[−0.52, 0.01]	−2.30	0.055	>0.250	0.81
AT vs. VB	0.08	[0.01, 0.15]	2.34	0.036	0.217	0.62	−0.15	[−0.39, 0.09]	−1.47	0.186	>0.250	0.52
AT vs. VR	0.01	[−0.07, 0.09]	0.26	>0.250	>0.250	0.07	−0.33	[−0.57, −0.09]	−3.25	0.014	0.084	1.15
VB vs. VF	−0.28	[−0.38, −0.18]	−6.18	<0.001	<0.001	1.65	−0.11	[−0.40, 0.18]	−0.87	>0.250	>0.250	0.31
VB vs. VR	−0.07	[−12, −0.02]	−20.89	0.013	0.077	0.77	−0.18	[−0.29, −0.07]	−3.85	0.006	0.038	1.36
VF vs. VR	0.21	[0.12, 0.31]	4.77	<0.001	0.002	1.27	−0.08	[−0.35, 0.20]	−0.65	>0.250	>0.250	0.23

*The results of the percentage of successful trials, the stability, the mean asynchrony, and the lag-1 autocorrelation (AC-1) are presented. (Note that when an ANOVA did not reveal a statistical effect of sequence type, post-hoc comparisons were not conducted and are leaved blank). Other conventions are as in Table 2*.

The stability results are illustrated in Figure 2B (mean values are listed in Table 2). A one-way repeated measures analysis of variance (ANOVA) with the factor sequence type (four sequence types) showed a statistical effect [*F*_(3, 42)_ = 21.35, *p* < 0.001, partial η^2^ = 0.60]. The *post-hoc* comparisons between sequence types are listed in Table 3, which revealed the following results. (1) Synchronization was much less stable for the VF sequence than for the AT and VB sequences (*p*_*corrected*_ < 0.05) and there was no statistical difference between the stabilities of the AT and VB sequences (*p*_*uncorrected*_> 0.250), replicating the improvement of synchronization performance by periodically moving visual stimuli (Gan et al., 2015; Iversen et al., 2015). (2) There was no statistical difference between the stabilities of the AT and VR sequences, although the stability was slightly higher for the AT than for the VR sequence (*p*_*uncorrected*_ > 0.250). This supported the suggestion that the speed characteristic is a critical factor contributing to the realism of a periodically moving visual stimulus and substantially improves synchronization (Hove et al., 2010, 2013a,b; Su, 2014; Gan et al., 2015). (3) As for the focus of Experiment 1, synchronization to the VR sequence was less stable than synchronization to the VB sequence (*p*_*corrected*_ < 0.05), indicating that the speed characteristic alone was not sufficient to explain the effect of the bouncing ball.

The mean asynchrony was statistically negative for the AT, VB, and VR sequences (*t*-test against zero: *p* < 0.05), and showed a marginal negativity for the VF sequence (*p* = 0.053) (Table 2). A one-way ANOVA with the factor sequence type (four sequence types) did not show a statistical effect [*F*_(3, 39)_ = 0.67, *p* > 0.250, partial η^2^ = 0.05].

Statistically negative AC-1 values were observed for the AT, VB, and VR sequences (*t*-test against zero: *p* < 0.05) but not for the VF sequence (*p* > 0.250) (Table 2), which suggested the involvement of error correction in synchronization to the auditory beat and the visual beat composed of periodically moving stimuli. A one-way ANOVA with the factor sequence type (four sequence types) showed a statistical effect [*F*_(3, 39)_ = 19.46, *p* < 0.001, partial η^2^ = 0.60] and the *post-hoc* comparisons between sequence types are listed in Table 3.

### Experiment 2

Experiment 2 was the same as Experiment 1 except that a short 300-ms IOI was used. The major concern of Experiment 2 was whether the stability difference between the VB and VR sequences could be larger in the 300-ms than in the 600-ms IOI condition.

**Figure 3:**
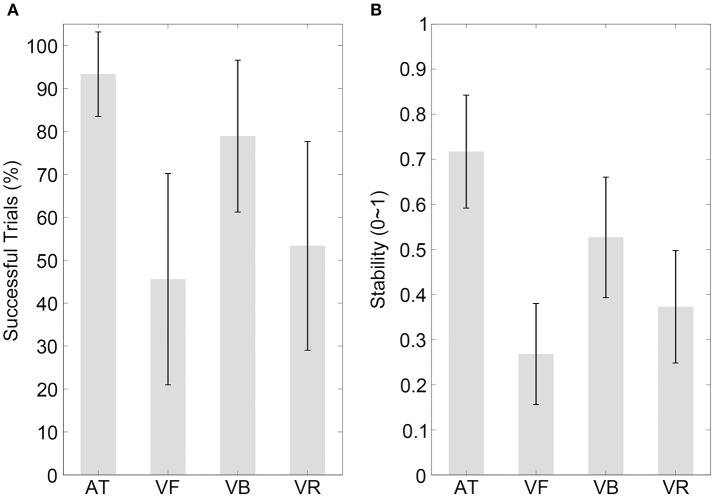
Results of experiment 2. **(A,B)** Show the percentages of successfully synchronized trials and the synchronization stabilities, respectively, for the four sequence types with a 300-ms IOI. Conventions are as in Figure 2.

The results of the percentage of successful trials are illustrated in Figure 3A (mean values are listed in Table 2). A one-way ANOVA with the factor sequence type (four sequence types) showed a statistical effect [*F*_(3, 42)_ = 7.98, *p* = 0.001, partial η^2^ = 0.36] and the *post-hoc* comparisons between sequence types are listed in Table 3.

The stability results are illustrated in Figure 3B (mean values are listed in Table 2). Consistent with previous reports (Gan et al., 2015), the stability decreased for all sequence types in the short 300-ms IOI condition, especially for the visual sequences. A one-way ANOVA with the factor sequence type (four sequence types) showed a statistical effect [*F*_(3, 42)_ = 21.31, *p* < 0.001, partial η^2^ = 0.60]. The *post-hoc* comparisons between sequence types are listed in Table 3, which revealed that synchronization to the VB sequence was more stable than synchronization to the VR sequence (*p*_*corrected*_ < 0.05). As for the focus of Experiment 2, this performance difference between the VB and VR sequences was larger in the 300-ms than in the 600-ms IOI condition (*Mean* = 0.12, *CI* = [0.05, 0.20], *t*_(14)_ = 3.45, *p* = 0.004, Cohen's *d* = 0.89). This result was hard to be interpreted by the compatibility factor; rather, it suggested that the speed characteristic made less contribution to the effect of the bouncing ball relative to other possible ecological factors, in the 300-ms than in the 600-ms IOI condition.

Moreover, consistent with previous findings (Repp, 2006; Gan et al., 2015), the difficulty of tapping in the 300-ms IOI condition was also reflected in the results of the mean asynchrony and AC-1. No statistically negative mean asynchrony was observed (*t*-test against zero: *p* > 0.250) (Table 2). A one-way ANOVA with the factor sequence type (four sequence types) showed a statistical effect [*F*_(3, 21)_ = 4.63, *p* = 0.030, partial η^2^ = 0.40] and the *post-hoc* comparisons between sequence types are listed in Table 3. The AC-1 was only statistically negative for the AT sequence (*p* = 0.025) (Table 2). A one-way ANOVA with the factor sequence type (four sequence types) showed a statistical effect [*F*_(3, 21)_ = 3.86, *p* = 0.042, partial η^2^ = 0.36] and the *post-hoc* comparisons between sequence types are listed in Table 3.

### Control experiments

In Experiments 1 and 2, for the same movement distance (0.92 cm for the 600-ms IOI or 0.77 cm for the 300-ms IOI), the ratio between the movement distance and the size of a periodically moving visual object was small for the bouncing ball (0.53 for the 600-ms IOI or 0.44 for the 300-ms IOI) whereas was large for the thin contracting ring (9.20 for the 600-ms IOI or 7.70 for the 300-ms IOI), which resulted in large discontinuities for the contracting ring, especially in the short 300-ms IOI condition. The ratio was manipulated in two control experiments using 600 and 300-ms IOIs. The major concern of the control experiments was whether the change of the ratio could explain the stability differences between the VB and VR sequences as observed in Experiments 1 and 2. Control experiment 1 increased the ratio by reducing the diameter of the bouncing ball. The ball diameter was either the same as that in Experiments 1 and 2 (1.74 cm), or was reduced by half (0.87 cm. The corresponding ratio was 1.06 for the 600-ms IOI or 0.89 for the 300-ms IOI). Control experiment 2 reduced the ratio by increasing the thickness of the contracting ring. The ring thickness was either the same as that in Experiments 1 and 2 (0.10 cm), or was increased to 0.87 cm (i.e., the half diameter of the bouncing ball. Correspondingly, the ratio was 1.06 for the 600-ms IOI or 0.89 for the 300-ms IOI, the same as the half-diameter ball), or was increased to 1.74 cm (i.e., the full diameter of the bouncing ball. Correspondingly, the ratio was 0.53 for the 600-ms IOI or 0.44 for the 300-ms IOI, the same as the full-diameter ball). For both the bouncing ball and the contracting ring, the effect of movement smoothness modulation was clear to the subjects as verbally reported. The prediction was: if the stability differences between the bouncing ball and contracting ring in Experiments 1 and 2 were related to the movement discontinuity, such stability differences should also be observed when the diameter of the ball or the thickness of the ring was manipulated.

**Figure 4:**
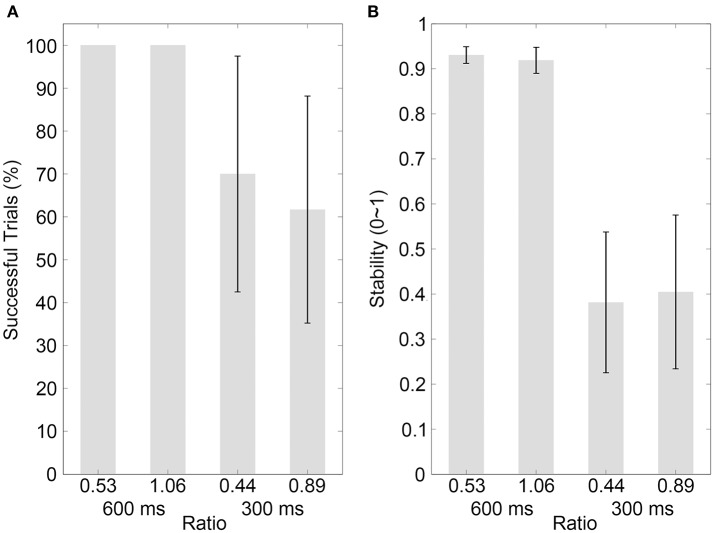
Results of control experiment 1. **(A,B)** Show the percentages of successfully synchronized trials and the synchronization stabilities, respectively, for the visual bouncing ball sequences with different movement-distance/object-size ratios in the 600 or 300-ms IOI condition. Conventions are as in Figure 2.

**Figure 5:**
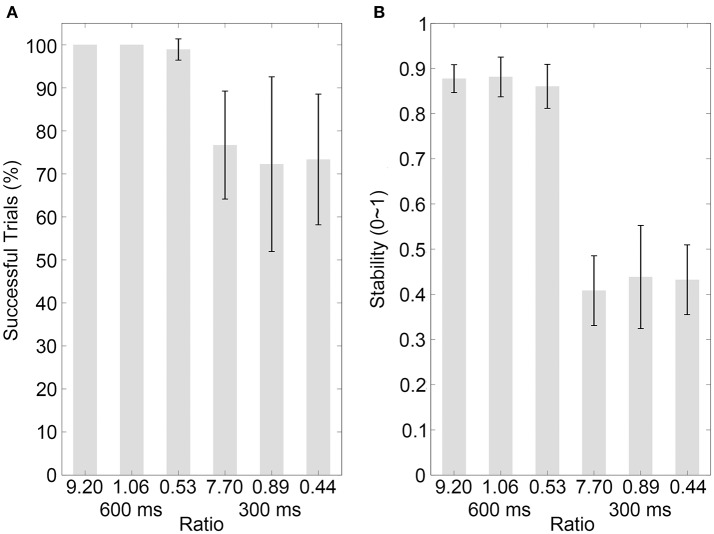
Results of control experiment 2. **(A,B)** Show the percentages of successfully synchronized trials and the synchronization stabilities, respectively, for the visual contracting ring sequences with different movement-distance/object-size ratios in the 600 or 300-ms IOI condition. Conventions are as in Figure 2.

The results of the percentage of successful trials in Control experiment 1 are illustrated in Figure 4A (mean values are listed in Table 4). A two-way ANOVA with factors of ratio (two movement-distance/object-size ratios) and IOI (600 and 300 ms) showed a statistical main effect of the factor IOI [*F*_(1, 9)_ = 8.65, *p* = 0.016, partial η^2^ = 0.49], but there was neither a statistical main effect of the ratio [*F*_(1, 9)_ = 2.65, *p* = 0.138, partial η^2^ = 0.23] nor a statistical interaction between the two factors [*F*_(1, 9)_ = 2.65, *p* = 0.138, partial η^2^ = 0.23]. For Control Experiment 2, the results are illustrated in Figure 5A (mean values are listed in Table 4). A two-way ANOVA with factors of ratio (three movement-distance/object-size ratios) and IOI (600 and 300 ms) showed a statistical main effect of the factor IOI [*F*_(1, 14)_ = 14.93, *p* = 0.002, partial η^2^ = 0.52], but there was neither a statistical main effect of the ratio [*F*_(1, 14)_ = 0.29, *p* > 0.250, partial η^2^ = 0.02] nor a statistical interaction between the two factors [*F*_(1, 14)_ = 0.21, *p* > 0.250, partial η^2^ = 0.02].

**Table 4:** Statistical information of synchronization measures in Control experiments 1 and 2.

	**Control experiment 1**					**Control experiment 2**			
	**Mean**	**95% CI**	***t*-value**	***p*-value**		**Mean**	**95% CI**	***t*-value**	***p*-value**
**SUCCESSFUL TRIALS (%)**									
0.53/600 ms	100.00	—	—	—	9.20/600 ms	100.00	—	—	—
1.06/600 ms	100.00	—	—	—	1.06/600 ms	100.00	—	—	—
0.44/300 ms	70.00	[42.58, 97.42]	5.78	<0.001	0.53/600 ms	98.89	[96.51, 101.27]	89.00	<0.001
0.89/300 ms	61.67	[35.27, 88.06]	5.29	0.001	7.70/300 ms	76.67	[64.19, 89.15]	13.18	<0.001
					0.89/300 ms	72.22	[51.98, 92.46]	7.65	<0.001
					0.44/300 ms	73.33	[58.21, 88.46]	10.40	<0.001
**STABILITY (0**~**1)**									
0.53/600 ms	0.93	[0.91, 0.95]	119.50	<0.001	9.20/600 ms	0.88	[0.85, 0.91]	62.56	<0.001
1.06/600 ms	0.92	[0.89, 0.95]	73.61	<0.001	1.06/600 ms	0.88	[0.84, 0.92]	43.60	<0.001
0.44/300 ms	0.38	[0.23, 0.54]	5.55	<0.001	0.53/600 ms	0.86	[0.81, 0.91]	38.61	<0.001
0.89/300 ms	0.40	[0.23, 0.57]	5.38	<0.001	7.70/300 ms	0.41	[0.33, 0.48]	11.44	<0.001
					0.89/300 ms	0.44	[0.32, 0.55]	8.29	<0.001
					0.44/300 ms	0.43	[0.36, 0.51]	12.09	<0.001
**MEAN ASYNCHRONY (RADIAN)**									
0.53/600 ms	−0.64	[−0.86, −0.42]	−6.85	<0.001	9.20/600 ms	−0.94	[−1.22, −0.65]	−7.14	<0.001
1.06/600 ms	−0.73	[−0.94, −0.51]	−7.92	<0.001	1.06/600 ms	−1.11	[−1.45, −0.78]	−7.36	<0.001
0.44/300 ms	0.45	[−0.18, 0.56]	1.41	>0.250	0.53/600 ms	−1.14	[−1.46, −0.81]	−7.61	<0.001
0.89/300 ms	0.19	[−0.30, 1.20]	1.23	0.202	7.70/300 ms	0.16	[−0.34, 0.67]	0.70	>0.250
					0.89/300 ms	0.46	[0.04, 0.88]	2.38	0.035
					0.44/300 ms	0.33	[−0.00, 0.66]	2.18	0.050
**AC-1**									
0.53/600 ms	−0.29	[−0.36, −0.22]	−9.50	<0.001	9.20/600 ms	−0.22	[−0.29, −0.14]	−6.16	<0.001
1.06/600 ms	−0.31	[−0.43, −0.19]	−6.12	<0.001	1.06/600 ms	−0.25	[−0.31, −0.19]	−9.30	<0.001
0.44/300 ms	0.09	[−0.05, 0.22]	1.55	>0.250	0.53/600 ms	−0.23	[−0.30, −0.17]	−7.80	<0.001
0.89/300 ms	−0.02	[−0.15, 0.11]	−0.29	0.166	7.70/300 ms	0.08	[−0.01, 0.18]	1.86	0.087
					0.89/300 ms	−0.01	[−0.13, 0.11]	−0.18	>0.250
					0.44/300 ms	0.03	[−0.10, 0.17]	0.54	>0.250

*Conditions in control experiments are indicated by ratio/IOI. Other conventions are as in Table 2*.

The stability results in Control experiment 1 are illustrated in Figure 4B (mean values are listed in Table 4). A two-way ANOVA with factors of ratio (two movement-distance/object-size ratios) and IOI (600 and 300 ms) showed a statistical main effect of the factor IOI [*F*_(1, 9)_ = 67.10, *p* < 0.001, partial η^2^ = 0.88], but there was neither a statistical main effect of the ratio [*F*_(1, 9)_ = 0.13, *p* > 0.250, partial η^2^ = 0.01] nor a statistical interaction between the two factors [*F*_(1, 9)_ = 1.90, *p* = 0.202, partial η^2^ = 0.17]. For Control experiment 2, the results are illustrated in Figure 5B (mean values are listed in Table 4). A two-way ANOVA with factors of ratio (three movement-distance/object-size ratios) and IOI (600 and 300 ms) showed a statistical main effect of the factor IOI [*F*_(1, 14)_ = 141.09, *p* < 0.001, partial η^2^ = 0.91], but there was neither a statistical main effect of the ratio [*F*_(2, 28)_ = 0.35, *p* > 0.250, partial η^2^ = 0.03] nor a statistical interaction between the two factors [*F*_(2, 28)_ = 0.57, *p* > 0.250, partial η^2^ = 0.04]. Therefore, these results were unlikely to support an effect of the movement discontinuity.

The mean values of the mean asynchrony are listed in Table 4. For Control experiment 1, a two-way ANOVA with factors of ratio (two movement-distance/object-size ratios) and IOI (600 and 300 ms) showed a statistical main effect of the factor IOI [*F*_(1, 7)_ = 18.36, *p* = 0.004, partial η^2^ = 72], but there was neither a statistical main effect of the ratio [*F*_(1, 7)_ = 1.56, *p* > 0.250, partial η^2^ = 0.18] nor a statistical interaction between the two factors [*F*_(1, 7)_ = 0.45, *p* > 0.250, partial η^2^ = 0.06]. For Control Experiment 2, a two-way ANOVA with factors of ratio (three movement-distance/object-size ratios) and IOI (600 and 300 ms) showed a statistical main effect of the factor IOI [*F*_(1, 12)_ = 43.63, *p* < 0.001, partial η^2^ = 0.78], but there was neither a statistical main effect of the ratio [*F*_(2, 24)_ = 0.26, *p* > 0.250, partial η^2^ = 0.02] nor a statistical interaction between the two factors [*F*_(2, 24)_ = 3.74, *p* = 0.055, partial η^2^ = 0.24].

The mean values of the AC-1 are listed in Table 4. For Control experiment 1, a two-way ANOVA with factors of ratio (two movement-distance/object-size ratios) and IOI (600 and 300 ms) showed a statistical main effect of the factor IOI [*F*_(1, 7)_ = 22.45, *p* = 0.002, partial η^2^ = 0.76], but there was neither a statistical main effect of the ratio [*F*_(1, 7)_ = 2.51, *p* = 0.157, partial η^2^ = 0.26] nor a statistical interaction between the two factors [*F*_(1, 7)_ = 3.08, *p* = 0.123, partial η^2^ = 0.31]. For Control Experiment 2, a two-way ANOVA with factors of ratio (three movement-distance/object-size ratios) and IOI (600 and 300 ms) showed a statistical main effect of the factor IOI [*F*_(1, 12)_ = 19.29, *p* = 0.001, partial η^2^ = 0.62], but there was neither a statistical main effect of the ratio [*F*_(2, 24)_ = 2.06, *p* = 0.153, partial η^2^ = 0.15] nor a statistical interaction between the two factors [*F*_(2, 24)_ = 0.56, *p* > 0.250, partial η^2^ = 0.05].

### Additional stability analyses of successful trials

**Figure 6:**
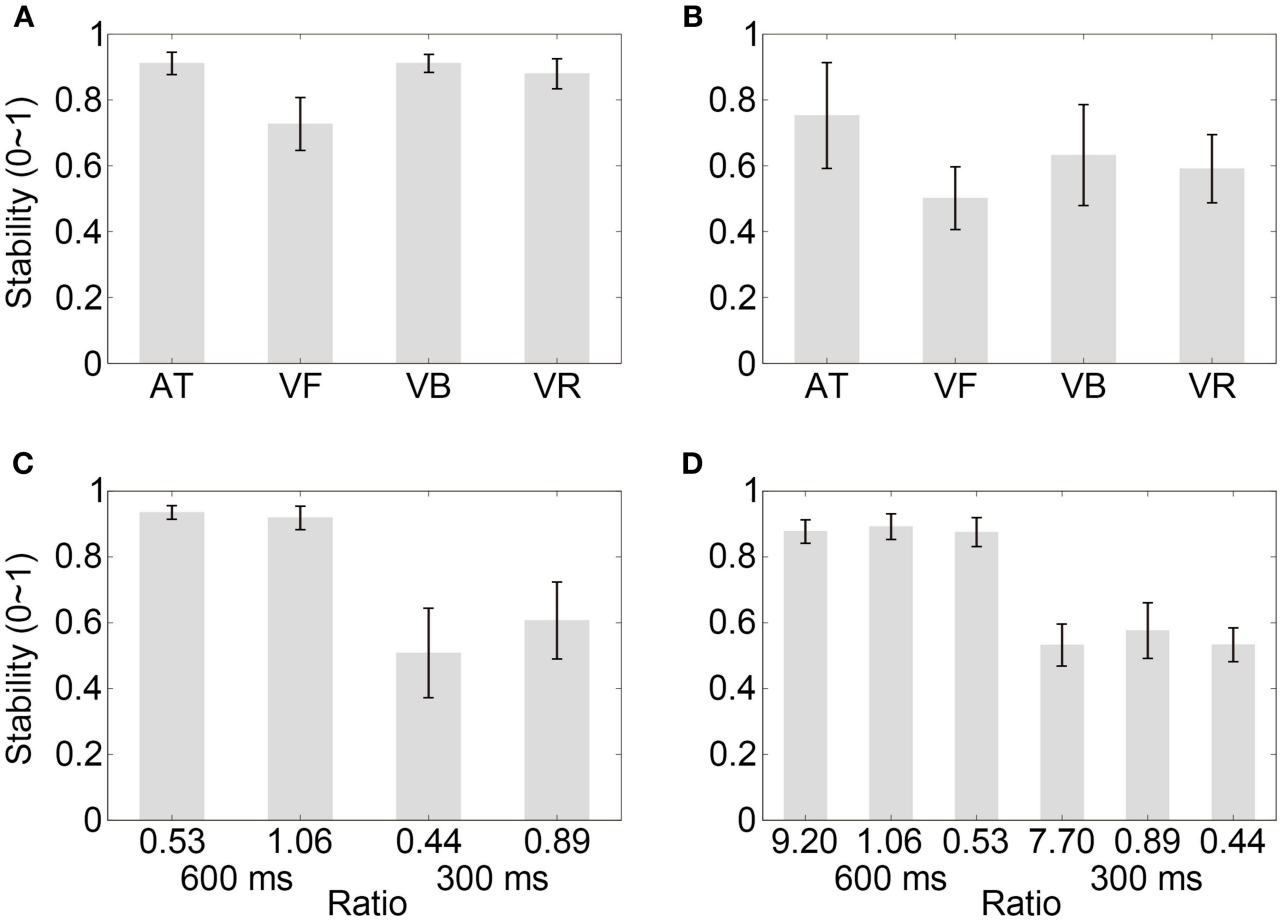
Stability results for successful trials. **(A–D)** Show the results of Experiment 1, Experiment 2, Control experiment 1, and Control experiment 2, respectively. Conventions are as in Figure 2.

In the above analyses, both successful and unsuccessful trials were included in the stability analyses. Here, the stability analyses containing only successful trials were performed and showed consistent results (Figure 6). For Experiment 1 (mean values are listed in Table 5 for all experiments), a one-way repeated measures analysis of variance (ANOVA) with the factor sequence type (four sequence types) showed a statistical effect [*F*_(3, 39)_ = 26.48, *p* < 0.001, partial η^2^ = 0.67]. The *post-hoc* comparisons between sequence types are listed in Table 6 for Experiments 1 and 2. For Experiment 2, a one-way ANOVA with the factor sequence type (four sequence types) showed a statistical effect [*F*_(3, 21)_ = 7.04, *p* = 0.009, partial η^2^ = 0.50]. For Control Experiment 1, A two-way ANOVA with factors of ratio (two movement-distance/object-size ratios) and IOI (600 and 300 ms) showed a statistical main effect of the factor IOI [*F*_(1, 7)_ = 66.94, *p* < 0.001, partial η^2^ = 0.91], and there was no statistical main effect of the ratio [*F*_(1, 7)_ = 4.61, *p* = 0.069, partial η^2^ = 0.40]. The interaction between the two factors was statistically significant [*F*_(1, 7)_ = 11.72, *p* = 0.011, partial η^2^ = 0.63], which was related to a stability difference between the two movement-distance/object-size ratios in the 300- ms IOI condition [*t*_(7)_ = 2.80, *p* = 0.027, Cohen's *d*_*z*_ = 0.99]. For Control Experiment 2, a two-way ANOVA with factors of ratio (three movement-distance/object-size ratios) and IOI (600 and 300 ms) showed a statistical main effect of the factor IOI [*F*_(1, 12)_ = 162.31, *p* < 0.001, partial η^2^ = 0.93], and there was neither a statistical main effect of the ratio [*F*_(2, 24)_ = 1.45, *p* > 0.250, partial η^2^ = 0.11] nor a statistical interaction between the two factors [*F*_(2, 24)_ = 0.27, *p* > 0.250, partial η^2^ = 0.02].

**Table 5:** Statistical information of the stability for successful trials in all Experiments.

	**Mean**	**95% CI**	***t* value**	***p* value**		**Mean**	**95% CI**	***t* value**	***p* value**
**Experiment 1**					**Experiment 2**				
AT	0.91	[0.88, 0.94]	61.43	<0.001	AT	0.75	[0.59, 0.91]	11.19	<0.001
VF	0.73	[0.65, 0.81]	19.89	<0.001	VF	0.50	[0.41, 0.60]	12.59	<0.001
VB	0.91	[0.89, 0.94]	77.31	<0.001	VB	0.63	[0.48, 0.78]	9.89	<0.001
VR	0.88	[0.84, 0.92]	43.04	<0.001	VR	0.59	[0.49, 0.69]	13.63	<0.001
**Control experiment 1**					**Control experiment 2**				
0.53/600 ms	0.92	[0.88, 0.95]	62.90	<0.001	9.20/600 ms	0.88	[0.84, 0.91]	55.43	<0.001
1.06/600 ms	0.93	[0.92, 0.95]	116.75	<0.001	1.06/600 ms	0.89	[0.85, 0.93]	52.44	<0.001
0.44/300 ms	0.61	[0.49, 0.72]	12.36	<0.001	0.53/600 ms	0.88	[0.83, 0.92]	44.87	<0.001
0.89/300 ms	0.51	[0.37, 0.64]	8.92	<0.001	7.70/300 ms	0.53	[0.47, 0.59]	18.55	<0.001
					0.89/300 ms	0.58	[0.49, 0.66]	15.12	<0.001
					0.44/300 ms	0.53	[0.48, 0.58]	23.01	<0.001

*Conventions are as in Tables 2, 4*.

**Table 6:** *Post-hoc* comparisons of the stability for successful trials between sequence types in Experiments 1 and 2.

	**Experiment 1**						**Experiment 2**					
	**Mean**	**95% CI**	***t-*value**	***p-*value**	***p_*corrected*_* value**	**Cohen's *d_*z*_* value**	**Mean**	**95%CI**	***t-*value**	***p-*value**	***p_*corrected*_* value**	**Cohen's *d_*z*_* value**
AT vs. VF	0.18	[0.11, 0.26]	5.22	< 0.001	0.001	1.40	0.25	[0.07, 0.43]	3.28	0.014	0.081	1.16
AT vs. VB	0.00	[−0.03, 0.03]	0.01	>0.250	>0.250	0.00	0.12	[−0.02, 0.26]	1.99	0.087	>0.250	0.70
AT vs. VR	0.03	[0.00, 0.06]	2.10	0.056	>0.250	0.56	0.16	[0.01, 0.31]	2.51	0.040	0.242	0.89
VB vs. VF	0.18	[0.12, 0.25]	6.33	< 0.001	< 0.001	1.69	0.13	[0.02, 0.24]	2.82	0.026	0.154	1.00
VB vs. VR	0.03	[0.01, 0.06]	2.89	0.013	0.076	0.77	0.04	[−0.05, 0.13]	1.10	>0.250	>0.250	0.39
VF vs. VR	−0.15	[−0.22, −0.09]	−5.00	< 0.001	0.001	1.34	−0.09	[−0.17, 0.00]	−2.50	0.041	0.245	0.88

*Conventions are as in Table 3*.

## Discussion

The present study investigated synchronization to four types of isochronous sequences with a 600 or 300-ms IOI: the auditory tone sequence, the visual flashing ball sequence, the visual bouncing ball sequence, and the visual contracting ring sequence. The contracting ring sequence had the same varying speed and thus the same speed characteristic as the bouncing ball sequence, but lacked other possible factors associated with the ecological relevance of the bouncing ball sequence. In the 600-ms IOI condition, synchronization was stable for the auditory tone, bouncing ball, and contracting sequences but not for the flashing ball sequence; and synchronization was more stable for the bouncing ball than for the contracting ring sequence. The stability decreased for all sequence types in the difficult 300-ms IOI condition, and the stability difference between the bouncing ball and contracting ring sequences was larger in the 300-ms than in the 600-ms IOI condition.

The superiority of the auditory over visual modality in beat synchronization is one of the best-known results in studies of sensorimotor synchronization (Repp, 2006). Recent findings of the improvement of synchronization by periodically moving visual stimuli suggest a modality non-specific view of beat synchronization (Hove et al., 2010, 2013a; Gan et al., 2015; Iversen et al., 2015), and ecological relevance of periodically moving visual stimuli has been proposed to play an essential role in the synchronization improvement (Repp and Penel, 2004; Hove et al., 2013a). Whereas the speed characteristic of periodically moving visual stimuli has been suggested to be the most critical factor in improving synchronization to a visual beat (Hove et al., 2010, 2013a,b; Su, 2014; Gan et al., 2015), the ecological relevance could be related to multiple factors and the relative contributions of the speed characteristic and other possible factors remain unknown. The present results suggested that factors other than the speed characteristic are required to interpret the synchronization improvement by periodically moving visual stimuli, especially in difficult tapping conditions.

An ecological bouncing ball with a uniformly varying speed (Gan et al., 2015) was adopted in the present study. One limitation of the present study should be pointed out, which rooted in the difficulty in identifying how many possible factors could be related to the ecological relevance of the bouncing ball, as described in the Introduction. Accordingly, in the present study the ecological factors other than the speed characteristic were vaguely defined. While the contracting ring had the same speed characteristic as the bouncing ball, it is hard to precisely describe the aspects in which the two stimulus types differed. Given the limitation, however, it was also worth emphasizing that the contracting ring lacked factors that are apparently associated with the ecological relevance of the bouncing ball. Regarding the current purpose of investigating the relative contributions of the speed characteristic and other possible factors to the overall effect of ecological relevance in improving synchronization to a visual beat, the present experimental design would be appropriate for this investigation aim, despite of the vaguely defined other possible factors. Therefore, the present study represents a pilot attempt to address the overall effect of ecological relevance in improving synchronization to a visual beat, and future research may devise better solutions to define possible ecological factors and examine the overall ecological effect. Moreover, it would be noted that the present study was not designed to investigate whether a specific ecological factor such as the speed characteristic is important in improving synchronization performance. For this aim, the experimental design would be to vary the specific factor while keeping other factors constant, as tried in the study of Gan et al. (2015) in which a uniformly varying speed and a sinusoidally varying speed were compared and other factors of the bouncing ball were unchanged. Such a design would not be appropriate for the current investigation of the relative contributions of the speed characteristic and other possible factors.

As has been discussed in the Introduction, periodically moving visual stimuli vs. static visual stimuli lead to a collision (Hove et al., 2010, 2013a) and periodically moving visual stimuli with a varying vs. constant speed lead to a peak speed at the collision point (Hove et al., 2010; Su, 2014; Gan et al., 2015; Iversen et al., 2015). Therefore, the characteristic of the speed is related to the characteristic of the collision, and it remains to be further addressed how to dissociate the effect of the speed characteristic from that of the collision characteristic. In the present study the speed characteristic was the same for the bouncing ball and the contracting ring. The collision characteristic, however, could differ between the two stimulus types. The sense of a collision may be stronger for the bouncing ball than for the contracting because the former rebounded on a surface and the latter rebounded on a point. Such collision characteristics would contribute to the ecological relevance of the bouncing ball, and the differences in the collision characteristics between the bouncing ball and the contracting ring would belong to the “other possible factors” which made the bouncing ball more ecological than the contracting ring, as discussed above.

The movement direction of the periodically moving visual stimuli and the movement direction of the tapping finger were compatible for the bouncing ball and were incompatible for the contracting ring. While we suggest that this compatibility factor did not change in the 600 and 300-ms IOI conditions, it would be further addressed that whether the incompatibility could be related to the tempo or tapping difficulty (e.g., possible ceiling effect in the 600-ms IOI condition) and thus becomes more detrimental in the 300-ms IOI condition. In the control experiments, the movement smoothness of the bouncing ball and the contracting ring was varied by modulating the ratio between the movement distance and the object size (the diameter of the ball or the thickness of the ring), and the results showed that the movement smoothness did not have a statistical effect on synchronization performance. In Gan et al. (2015), the movement smoothness of the bouncing ball in the 600-ms IOI condition was improved by using a higher- vs. lower-resolution computer screen, and a statistical effect of the movement smoothness was also not observed. The lack of an effect of the movement smoothness on synchronization to moving visual stimuli was a surprising result given that the movement discontinuity of the thin contracting ring in the 300-ms IOI condition was clear to the subjects, which would be further addressed.

It deserves to be mentioned that the design of the periodically contracting ring has practical significance for the investigation of neurophysiological mechanisms of beat perception and synchronization. Beat perception and synchronization have been hypothesized to be underlain by the entrainment of neuronal populations resonating at the beat frequency (Large and Jones, 1999; Large and Snyder, 2009). The resonance theory has been supported by electroencephalography (EEG) data that showed steady-state evoked potential (SSEP) activity at the beat frequency when listening and tapping to auditory beats (Nozaradan et al., 2011, 2013). The finding of synchronization improvements by periodically moving visual stimuli suggests a modality non-specific view of beat perception and synchronization (Hove et al., 2010, 2013a; Gan et al., 2015; Iversen et al., 2015), and the employment of such periodically moving visual stimuli has been proposed to be an important utility for investigation of the neural substrates of beat perception and synchronization (Hove et al., 2013a). However, for EEG research, a stimulus periodically moving along a specific spatial direction at the beat frequency (e.g., the bouncing ball or the moving bar that is typically used in beat synchronization studies) would induce eye movements at the beat frequency, which would lead to serious rhythmic EEG noises. Such rhythmic EEG noises at the beat frequency are hard to be distinguished from the to-be-investigated rhythmic EEG signals associated with beat perception and synchronization. We suggest that this eye movement issue is not a concern for the currently designed contracting ring because of its inward-outward movement, as has been well established in fMRI retinotopic mapping studies in which a contracting ring serves to map the retinotopic extent of visual areas (Sereno et al., 1995).

At last, it should be emphasized that for synchronization improvement by periodically moving visual stimuli as compared to visual flashes, the present study focused on ecological factors that referred to the perceptual attributes of the stimuli, such as the speed characteristic (Hove et al., 2010, 2013a,b; Su, 2014), the movement smoothness of stimuli (Gan et al., 2015), and the collision characteristic (Hove et al., 2010; Su, 2014; Iversen et al., 2015). Accordingly, the contracting ring was designed to investigate relative contributions of the speed characteristic and other possible perceptual factors. Besides perceptual factors, factors that are more related to action could also contribute to the synchronization improvement. One example of such action factors is the direction of hand movement, and the interaction (i.e., compatibility) between stimulus movement direction and hand movement direction has been investigated by Hove et al. (2010) [also addressed in Gan et al. (2015) and the present study]. Also note that the effect of the compatibility factor may be related to proprioception, which refers to the sense of body (particularly the limb) position and movement. For example, Weeks et al. (2017) showed that the proprioceptive acuity is better when the movement of a visual cursor is orthogonal to than in line with hand movement. The role of proprioceptive sensitivity in beat synchronization remains to be further examined. Because the present study focused on investigation of perceptual factors, the compatibility factor was addressed as a confounding factor. The interaction between perceptual and action factors would be further examined via, e.g., varying the directions of stimulus moment and hand movement in a more systematical manner.

In summary, the present study designed a periodically contracting ring that had the same speed characteristic as the ecological bouncing ball but lacked other possible factors associated with the ecological relevance of the periodically bouncing ball. Beat synchronization was more stable for the bouncing ball than for the contracting ring, and this stability difference was greater in the 300-ms than in the 600-ms IOI tapping condition. The finding provides new insights into how the speed characteristic and other possible ecological factors improve synchronization to a visual beat consisting of periodically moving stimuli.

## Data availability

The data generated during and/or analyzed during the current study are available from the corresponding author on reasonable request.

## Author contributions

XW conceived the research. YH and XW designed the research. YH performed Experiments 1 and 2, and Control experiment 1. YH and LG performed Control experiment 2. YH and LG analyzed the data. JY participated in the design of Control experiment 1. SZ was involved in the discussion of the data. XW wrote the manuscript. All authors commented on and edited the manuscript.

### Conflict of interest statement

The authors declare that the research was conducted in the absence of any commercial or financial relationships that could be construed as a potential conflict of interest. The reviewer AC and handling editor declared their shared affiliation.
